# A gene regulatory motif that generates oscillatory or multiway switch outputs

**DOI:** 10.1098/rsif.2012.0826

**Published:** 2013-02-06

**Authors:** Jasmina Panovska-Griffiths, Karen M. Page, James Briscoe

**Affiliations:** 1 Social and Mathematical Epidemiology Group, London School of Hygiene and Tropical Medicine, Faculty of Public Health and Policy, 15–17 Tavistock Place, London WC1H 9SH, UK; 2 Department of Mathematics, University College London, Gower Street, London WC1E 6BT, UK; 3 MRC National Institute for Medical Research, The Ridgeway, Mill Hill, London NW7 1AA, UK

**Keywords:** Sonic Hedgehog, neural tube, morphogen, multistate switch, oscillations, gene regulatory network

## Abstract

The pattern of gene expression in a developing tissue determines the spatial organization of cell type generation. We previously defined regulatory interactions between a set of transcription factors that specify the pattern of gene expression in progenitors of different neuronal subtypes of the vertebrate neural tube. These transcription factors form a circuit that acts as a multistate switch, patterning the tissue in response to a gradient of Sonic Hedgehog. Here, by simplifying aspects of the regulatory interactions, we found that the topology of the circuit allows either switch-like or oscillatory behaviour depending on parameter values. The qualitative dynamics appear to be controlled by a simpler sub-circuit, which we term the AC–DC motif. We argue that its topology provides a natural way to implement a multistate gene expression switch and we show that the circuit is readily extendable to produce more distinct stripes of gene expression. Our analysis also suggests that AC–DC motifs could be deployed in tissues patterned by oscillatory mechanisms, thus blurring the distinction between pattern-formation mechanisms relying on temporal oscillations or graded signals. Furthermore, during evolution, mechanisms of gradient interpretation might have arisen from oscillatory circuits, or vice versa.

## 1. Introduction

The formation of functioning, architecturally complex tissues during embryonic development relies on the spatially and temporally organized production of multiple distinct cell types. Understanding how this is achieved requires insight into the underlying molecular mechanisms. In broad terms, the transcriptional programme of a cell determines cellular identity. Thus, the spatial arrangement of the transcriptional programmes operating within the developing tissue determines the pattern of cell differentiation. In order for these programmes to be spatially organized a source of positional information to apprise cells of their location and a mechanism to convert this information into the appropriate transcriptional programme are required.

Several mechanisms have been identified. One strategy exploits oscillations that produce regular transitions in gene expression. Such oscillations, when applied across a growing field of cells, generate repeated domains of gene expression. A well-established example of this is somitogenesis, which divides vertebrate mesoderm into reiterated blocks of tissue, arrayed along the anterior–posterior axis [1]. A second strategy involves secreted molecules, termed morphogens, which establish signalling gradients across a field of cells. In simple terms, the spatial metric could be provided by the concentration of the morphogen. However, it has become apparent that the duration of signalling can also contribute to the positional identity of a cell [2,3]. Both oscillatory and morphogen mechanisms represent biological examples of multistate switches in which a single input signal (time and morphogen, respectively) produces multiple discrete outputs. However, the mechanisms by which these multistate switches are implemented remain poorly understood. Moreover, it remains unclear whether there are mechanisms in common between oscillatory and morphogen strategies of pattern formation.

To address these questions, we have focused on a specific example of pattern formation in which the secreted protein Sonic Hedgehog (Shh) patterns ventral regions of the vertebrate neural tube. In this tissue, Shh controls a multistate switch that establishes the distinct identities of neural progenitors that generate the distinct differentiated neuronal subtypes [4]. Shh is secreted from the notochord, which underlies the ventral neural tube, and the floor plate, located at the ventral midline of the neural tube, and diffuses to form a ventral-to-dorsal gradient. In response to this Shh gradient, progenitors in the ventral neural tube regulate the expression of a set of transcription factors (TFs); these include the homeobox proteins Pax6 and Nkx2.2 and the basic helix–loop–helix protein Olig2. The expression of these three TFs distinguishes discrete domains of progenitors that generate the three most ventral neuronal subtypes. The spatial pattern of expression depends on graded Shh signalling and, *in vitro*, high concentrations and longer durations of Shh activate Nkx2.2 compared with Olig2, whereas Pax6 is expressed in the absence of Shh signalling [5,6].

Shh signalling acts via an intracellular signal transduction pathway that culminates in the regulation of Gli TFs (Gli1, 2 and 3), a family of zinc finger containing transcriptional effectors [7,8]. Exposure of cells to Shh results in a net increase in the transcriptional activator function of Gli proteins. Gain- and loss-of-function studies indicate that most, if not all, the activities of Shh are transduced by Gli activity. Consistent with involvement of graded Shh signalling in their induction, Gli activity is necessary for the expression of Olig2 and Nkx2.2. Moreover, increased durations and higher levels of Gli activity are needed for the expression of Nkx2.2 than Olig2. In addition, Pax6, Olig2 and Nkx2.2 are part of a gene regulatory circuit that comprises a set of cross-repressive interactions between the TFs. Specifically, Pax6 and Nkx2.2 cross-repress each other's expression, as do Olig2 and Nkx2.2. Additionally, Olig2 represses Pax6 expression [9]. These cross-regulatory interactions have been proposed to be important for interpretation of Shh signalling and the generation of the discrete switches in gene expression that characterize the distinct progenitor domains. Here, we examine this idea and study the features of the gene regulatory circuit that allow for the interpretation of a graded signal. We further address whether the regulatory logic of this circuit can confer other properties on the response of the individual genes in the circuit.

The nonlinear behaviour exhibited by the Shh regulation of the TFs Pax6, Olig2 and Nkx2.2 is characteristic of many gene regulatory networks. In particular, the presence of multiple feedback loops make these networks difficult to intuitively understand simply by analysing molecular and genetic experiments. Mathematical modelling provides a convenient method to integrate such networks into a single coherent conceptual framework that allows for the elucidation of the logic and key principles of the circuit [10,11]. Sets of parameters for which the system exhibits biologically plausible behaviour can be determined and the presence of alternative behaviours and any emergent properties can be investigated.

Many mathematical models for gene regulatory networks have been developed (reviewed in Smolen *et al.* [12,13] and Hasty *et al.* [14]). A common approach is to describe the changing level of expression of each gene using ordinary differential equations (ODEs). In this way, a network is described as a dynamical system comprising a set of linked ODEs. An advantage of this approach is that a large amount of mathematical theory is available that allows these systems to be explored (e.g. stability analysis, bifurcation analysis, perturbations methods) giving an in-depth understanding of the system. For example, Tyson & Othmer [15], extending the work of Goodwin [16], used this approach to explore feedback loops in biochemical pathways with arbitrarily many components, determining conditions for the existence and stability of steady states and periodic solutions. The repressive loops in their system did not admit multistability. Subsequently, Cherry & Adler [17] considered more general models of two mutually repressive proteins and found conditions for bistability, which allowed the system to behave as a switch. Saka & Smith [18] demonstrated how this could be exploited to produce a morphogen response for two TFs. Smith [19] analysed generic *N*-species cyclic networks in which genes were connected by repressive interactions. This suggested qualitatively distinct behaviours depending on whether *N* was even or odd: when *N* was even, the system behaved as if regulated by positive feedback and had multiple steady states. In contrast, when *N* was odd the system exhibited periodic oscillations [19]. Similarly, Yang *et al.* [20] demonstrated how a three-gene network could generate oscillations by two different mechanisms.

A limitation of modelling approaches using ODEs is that, with the exception of the simplest systems, it is usually not possible to determine analytical solutions and numerical analysis can be computationally expensive. For these reasons, hybrid methods, which combine ODEs with aspects of Boolean algebra, have been deployed. With this framework, Boolean on–off switches represent the regulatory events that are characterized by sharp thresholds, whereas continuous input–output relations model the remaining events. This approach has been successfully applied to the analysis of the *E. coli*–phage lysis–lysogeny switch [21]. A class of hybrid model are piecewise linear models, which were pioneered by Glass & Kauffman [22] (see also [23,24]). The hybrid approach has a computational advantage over systems of ODEs and is more readily amenable to extensive numerical simulations and analysis.

**Figure 1.**
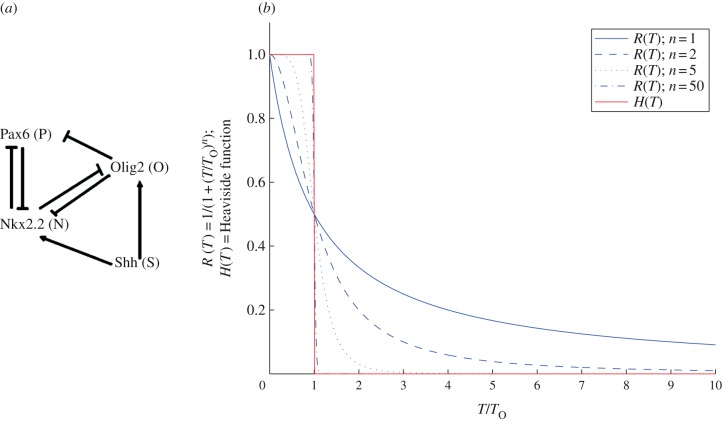
(*a*) Diagram of the gene circuit controlling the specification of neural progenitor domains in the vertebrate neural tube. The diagram shows the interactions between the morphogen Shh and the three TFs Pax6, Olig2 and Nkx2.2. (Pointed arrowheads indicate induction, while blunt arrowheads indicate repression.) (*b*) An illustration of how Hill functions approach a Heaviside (step) function, as the Hill coefficient tends to infinity. *T* represents the concentration of a repressor. The blue lines show repressive Hill functions, *R*(*T*), with Hill coefficients 1, 2, 5 and 50. The red line shows a repressive Heaviside function, *H*(*T*).

In this paper, we use a simple four-node circuit depicted in figure 1*a* to study the logic of the interplay between Shh signalling and the cross-repressive properties of the TFs Pax6, Olig2 and Nkx2.2. This network contains a subnetwork (figure 7*b*(ii)), which we term the ‘AC–DC signalling motif’. We use this motif to show how a relatively simple mathematical framework can reveal novel principles in the strategies by which tissues are patterned. In §2, we formulate a simplified version of the mathematical model presented in [9]. This model falls into the category of hybrid model described above. We explore the model analytically in §3. Numerical simulations of the reduced system verify our analysis and we show how the results compare to those from the full model in §4. The analysis indicates that the same motif can be used to interpret a temporal and spatial morphogen gradient and to generate oscillatory patterns. We find that the subnetwork, mentioned above, controls the qualitative dynamics of the network operating in the neural tube and, in §5, we propose that it represents a novel regulatory motif. In §6, we suggest that this motif is a natural one to give rise to three stripes of gene expression (here corresponding to three neural progenitor domains). We consider how the motif is likely to be generalized in a system with more than three stripes. We discuss our results in §7.

## 2. Model formulation

During neural tube patterning, Shh is secreted from the ventral pole of the neural tube. Shh signalling is mediated by Gli activity. In our model, we quantify this signalling with a parameter *S* which can be considered to be a measure of the Shh-induced Gli transcriptional activity. In the progenitor cells of the neural tube, Pax6 is expressed in the absence of Shh, whereas both Olig2 and Nkx2.2 are induced by Shh. Nkx2.2 and Olig2 cross-repress each other, as do Nkx2.2 and Pax6 [3,9]. In addition, Pax6 is repressed by Olig2. The regulatory architecture of this circuit is represented in figure 1*a*.

At this stage, we ignore stochastic effects involved in transcription, translation and decay and also transcriptional time delays. We model the cross-repressive interactions that link Pax6, Olig2 and Nkx2.2 together in a gene regulatory circuit as a dynamical system with an inducible signal. Our mathematical framework is a system of ODEs that describe the temporal evolution of the concentrations of the proteins Pax6, Olig2 and Nkx2.2, *P*(*t*), *O*(*t*) and *N*(*t*), respectively, at different cellular Gli activities (*S*). In [9], we assumed that the repressive effect of one TF on another could be represented by a declining Hill function in the production term.

The structure of this network contains positive and negative feedback loops between *P*, *O* and *N*. Although the architecture of the network looks relatively simple, the presence of these loops makes the system more complex than anticipated. To simplify the analysis, we let the repressions on Nkx2.2 and Olig2 become infinitely sharp (Hill coefficients tend to infinity), so that we replace Hill functions by Heaviside (i.e. on–off) functions (figure 1*b*). This approximation is in agreement with experimental observations that cross-repression between Olig2 and Nkx2.2 as well as repression of Pax6 on Nkx2.2 can be discrete, either on or off [25,26]. These studies show that Olig2 is completely inhibited by overexpression of Nkx2.2, and Nkx2.2 is completely inhibited by overexpression of Pax6. Furthermore, our recent work [9] provides evidence that Olig2 can inhibit Nkx2.2 induction. In contrast, the level of expression of Pax6 is spatially graded [26,27], so we retain the Hill function form for the repression on Pax6 by Olig2 and Nkx2.2. We arrive at the following equations:2.1
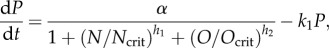
2.2
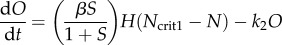
2.3
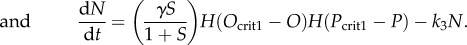


Degradation constants *k*_*i*_, where *i* = 1,2,3, describe the first-order decay of the proteins. We note that dilution owing to exponential growth may contribute to protein decay, but, since the key element we wish to explore is the transcriptional cross-repression, for simplicity, we ignore more complicated decay functions. The maximum rates of production of *P*, *O* and *N* are given by the positive constants *α*, *β* and *γ*, respectively. *H*(…) is the Heaviside function, that is, *H*(*x*) = 1 if *x* ≥ 0 and *H*(*x*) = 0 if *x* < 0. The values *P*_crit1_, *O*_crit1_ and *N*_crit1_ are the critical values of *P* at which *P* switches off *N* production, of *O* at which *O* switches off *N* production and of *N* at which *N* switches off *O* production, respectively. In the Hill function repression of *P*, 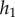 and 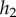 are Hill coefficients, which describe how sharply the repression of *P* depends on *N* concentration and *O* concentration, respectively. If *O* is absent, *N*_crit_ is the concentration of *N* at which *P* production is half-maximal. Likewise, if *N* is absent, *O*_crit_ is the concentration of *O* at which *P* production is half-maximal. We note that we have also investigated the effect of having cooperativity in the signal (*S*) mediated induction of *O* and *N* (i.e. Hill coefficients greater than one), but found that this does not have a marked effect on the system's behaviour. For simplicity, we therefore consider here only Michaelis–Menten forms of the induction. In addition, in mouse mutants lacking Pax6 and Olig2, Nkx2.2 expands up to the limit of the Olig2 domain observed in wild-type embryos. We therefore assume that the critical values of *S* at which *N* and *O* are induced are the same.

## 3. Steady states, linear stability analysis and model behaviour as Shh concentration is varied

In this section, we study the model (2.1)–(2.3). We determine analytical conditions on the model parameters that differentiate between the two biological scenarios: a tripartite expression pattern with a multistate switch in the expression of the TFs, and the presence of temporal oscillations in TF expression for some Shh levels. The quantitative (and potentially tunable) features of the gene circuit that allow us to differentiate these two cases will be the basis of the new signalling AC–DC motif we propose.

The simplification of using discrete Heaviside functions for the cross-repression functions on *O* and *N* allows the steady states of the dynamical system to be determined analytically. The system has three possible steady states, *B*_1_, *B*_2_ or *B*_3_ which vary with *S*:3.1
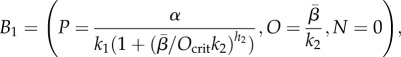
3.2
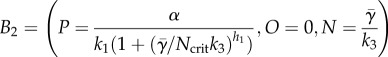
3.3
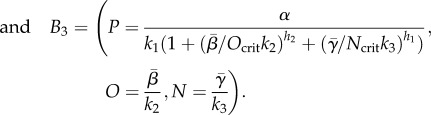


Here, 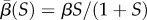 and 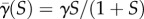, so that these vary with *S*. We note that for each state, *P* alone is expressed as 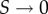.

It is straightforward to show that each of the states is stable where it exists. The criteria for existence of each state are as follows.

(a) *B*_1_ exists (and is stable) if and only if
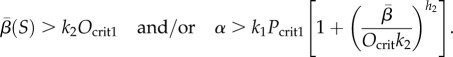
(b) *B*_2_ exists (and is stable) if and only if
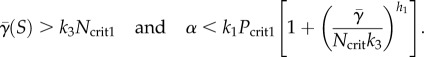
(c) *B*_3_ exists (and is stable) if and only if
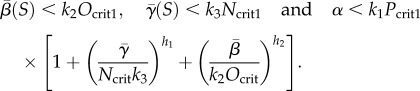

We note that states *B*_2_ and *B*_3_ cannot coexist, but, depending on the parameter values, state *B*_1_ can coexist with either of the other two states.

We now consider what happens to the system as the level of *S* is gradually increased (sufficiently gradually that the system may be considered always to be at steady state). To ensure that *N* is inactive at low values of *S*, we require *α* > *k*_1_*P*_crit1_. The system will hence evolve to state *B*_1_, which has zero *N* concentration. As the level of *S* is gradually increased, in order for this state to lose stability at some point (such that *N* is activated), we require 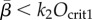. We note that in this simplified system, we only get realistic tripartite behaviour, without *N* being activated uniformly across the domain, if *O* never represses *N*. Intuitively, this is because, in the absence of *N*, the concentration of *O* increases with the level of *S*, so that if a cell, in which *O* is capable of repressing *N*, sees an increasing level of *S*, it is impossible for that repression to be switched off and therefore for *N* to be expressed.

It is worth pointing out that the condition that *O* never represses *N* is only a requirement because of the all-or-nothing nature of the repression in our system. We have shown [9] that the full model system can give rise to realistic tripartite behaviour even when *O* represses *N*. Most of the key qualitative behaviours of this full system, however, are displayed also by the system without the repression of *N* by *O*. Minor quantitative differences do exist with and without *O* repression of *N*. For example, if *O* represses *N*, then *N* expression is delayed in wild-type embryos relative to *O* mutants.

In order for *O* to be switched off at high values of *S*, we require *B*_2_ to become stable and hence 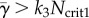. The inequalities necessary for the existence of *B*_2_ are then improved by further increasing the value *S*; therefore, once *B*_2_ is attained it cannot be left. In summary, if *N* is not to be induced by low levels of *S* and *O* is not to be active at high levels of *S*, then there are two possible routes through the steady states as the value of *S* increases:

(1) *B*_1_ → *B*_2_; or
(2) *B*_1_ → *B*_3_ → *B*_2_.

We set *P*_max_ = *α*/*k*_1_, *O*_max_ = *β*/*k*_2_ and *N*_max_ = *γ*/*k*_3_. Then for one of these biologically relevant routes to occur, necessary and sufficient conditions on the parameters are (for a derivation see appendix A)3.4
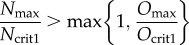
and3.5
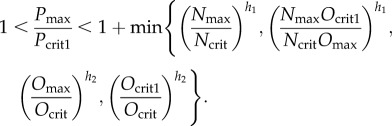


The first route is taken if3.6
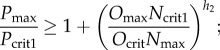
otherwise, the second route is adopted. In the second route, as *S* increases, there is an overlap in the existence of *B*_1_ and *B*_3_ and hence there is a range of Shh concentration for which the system is bistable and hysteresis is exhibited. This means that the level of *S* required to switch on *N* expression is higher than that required to maintain *N*, once it is activated.

In both routes, there are two possibilities for the transition to the state *B*_2_. In the first route, generically, as the level of *S* is increased, there is either an overlap in the existence of *B*_1_ with *B*_2_, or there is a gap. At a critical set of parameter values, there is a sharp transition into *B*_2_ from *B*_1_ at a fixed value of *S*. In the second route, there is either a direct switch (with no gap or overlap) in existence from *B*_3_ to *B*_2_, or there is a gap. In the cases where there is a gap in steady states, oscillations result. For levels of *S* that fall within this range, when *N* switches off *O*, it is not sufficient to keep *P* switched off. This allows *P* to rise, which in turn switches off *N*. Consequently, this allows the reactivation of *O* that then represses *P*, allowing the level of *N* to rise again. The process then repeats. The resulting dynamics are oscillatory, changing from *N* high to *P* high to *O* high and repeating. By contrast, in the case of an overlap or a direct switch in stability, when the levels of *N* rise and switch off *O*, the activity of *N* is capable of exerting sufficient repression on *P* to maintain its own expression.

In the first route, the condition for a gap in existence/stability between *B*_1_ and *B*_2_ is3.7
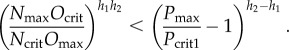
(In the case where *h*_1_ = *h*_2_, this simplifies to *N*_max_/*N*_crit_ < *O*_max_/*O*_crit_.)

In the second route, the condition for a gap in stability/existence between *B*_3_ and *B*_2_ is3.8
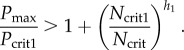


In summary, there are four distinct possibilities for the behaviour of the system as the level of *S* is increased, given that, for low values of *S*, *N* is absent, and, for high *S* values, *O* is absent.^1^ These possibilities are

(1) *N* off → *O* off,
(2) *N* off → *N* and *O* co-expressed → *O* off,
(3) *N* off → *N*, *O* and *P* oscillate → *O* off,
(4) *N* off → *N* and *O* co-expressed → *N*, *O* and *P* oscillate → *O* off.

The critical values of Shh concentration for which the transitions between behaviours occur are given in appendix B. In the first two possibilities, there is bistability around the transition to *N* on, and so the system displays hysteresis. We note that, had we not chosen parameters such that *N* is expressed at high *S* and *O* is not, we would have found parameter values for which the system oscillates for all *S* values above a threshold. When *S* is very low, there are no oscillations, since *P* alone is expressed. However, it is possible for oscillations to occur as *S* → ∞.

As we have mentioned, state *B*_1_ can coexist with state *B*_2_ or with state *B*_3_. In addition, the states are stable when they exist. Typically, we expect the system initially to have seen no Shh, so initially, *S* = 0 and so *N* = *O* = 0 and *P* = *α*/*k*_1_. If *S* is increased gradually (slowly compared with the degradation of *N*, *O* and *P*), then the system will remain in *B*_1_ until that state ceases to exist. By contrast, if a cell starts, for example, in state *B*_2_ (expressing Nkx2.2) and *S* is gradually decreased, the system will stay in *B*_2_ until that state ceases to exist. We have discussed (and experimentally verified) this hysteretic property of the system in [9].

## 4. Numerical results

In this section, we illustrate the predictions of the analysis in the previous section using numerical simulations in which there is a switch from *B*_1_ (no *N*) direct to *B*_2_ (no *O*) with increasing *S* or a switch from *B*_1_ via *B*_3_ (where *N* and *O* are co-expressed) to *B*_2_. We also illustrate how the transition to *B*_2_ can either be direct or can be separated from the previous steady state by a range of values of *S* for which the system displays oscillations.

The numerical simulations in this section were performed using the Matlab ODE solver ode45. For the bifurcation diagrams, the maximal and minimal values of *O* were then computed, once the system had converged to steady state or to oscillations. For the simulations of the Heaviside model, since the vector field is discontinuous, there are isolated points at which the solution is not continuously differentiable. We checked the results by comparing with numerical simulations of the Hill function model with very high Hill coefficients in the equations for *N* and *O*. The unstable steady states shown in figure 5 were computed numerically by solving the relevant algebraic equations.

**Figure 2.**
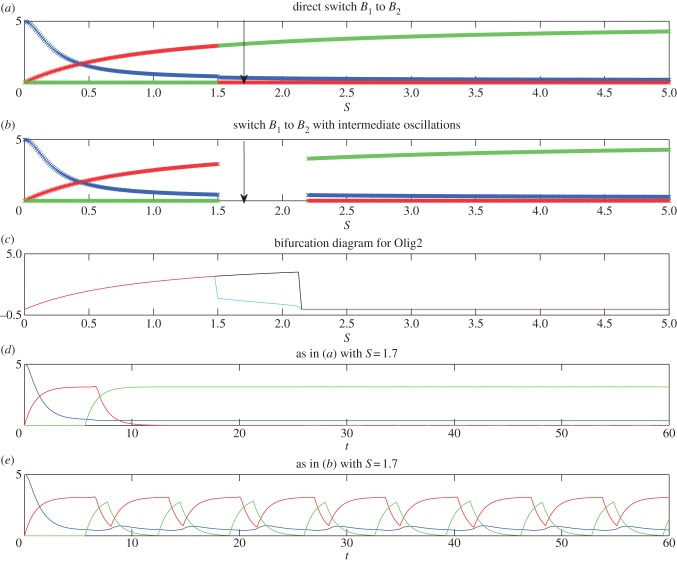
Numerical profiles of the simplified model, illustrating the existence of a sharp switch (*a*,*d*) or oscillations at intermediate S levels (*b*,*c*,*e*) in the expression profiles of Pax6 (blue), Olig2 (red) and Nkx2.2 (green) in the transition *B*_1_ → *B*_2_. Model parameters are: *α* = 5, *β* = 5, *γ* = 5, *h*_1_ = 2, *h*_2_ = 2, *O*_crit_ = 1, *P*_crit1_ = 0.5, *O*_crit1_ = 5, *N*_crit1_ = 2, *k*_1_ = 1, *k*_2_ = 1, *k*_3_ = 1 and in (*a*) and (*d*) *N*_crit_ = 0.9 and (*b*), (*c*) and (*e*) *N*_crit_ = 1.1. In (*a*) and (*b*), the profiles are generated at *t* = 60, at which time the profiles have converged to steady state (to within a tiny tolerance), if they ever will do so. If they do not, we simply leave a gap in the plot. In (*c*), we show the steady-state value (red line) or maximum (black line) and minimum (cyan line) values of *O* for parameters as in (*b*), once the system has converged either to steady state or to a limit cycle (we use *t* = 500), plotted against *S*. In (*d*) and (*e*), we plot time courses of *P*, *O* and *N* for parameters as in (*a*) and (*b*), respectively, and for fixed *S*. The values of *S* used in (*d*) and (*e*) are marked with arrows in (*a*) and (*b*), respectively.

Figure 2 shows numerical simulations for parameter values corresponding to possibilities (1) and (3) from the previous section, indicating how the levels of the three TFs vary as a function of *S* for two different parameter sets. We use *h*_1_ = *h*_2_ and in figure 2*a* the parameters satisfy *N*_max_/*N*_crit_ > *O*_max_/*O*_crit_, so that the transition to *B*_2_ is direct. By constrast, in figure 2*b* the parameters satisfy *N*_max_/*N*_crit_ < *O*_max_/*O*_crit_, so that there is an intermediate range of values of *S* for which there are temporal oscillations. Since, for these values of *S*, the system does not converge to steady state, the long-term values of *P*, *O* and *N* are not shown here. Instead, in figure 2*c*, we show either the stable steady state values of *O* (in red), or, once the system has converged to a limit cycle, the maximum (in black) and minimum (in cyan) values of *O*. In figure 2*d*, we illustrate the temporal behaviour of the model with parameter values as in figure 2*a*, for a level of *S* just above the transition from *B*_1_. The model predicts that first *P* is active, then *O* and finally *N*. In all of the numerical simulations of this section, we use initial conditions corresponding to the steady-state values in the absence of *S*, i.e. *P* = *α*/*k*_1_, *O* = *N* = 0. In figure 2*e*, we illustrate the temporal behaviour of the model with parameter values as in figure 2*b*, for a level of *S* just above the transition from *B*_1_. Here, temporal oscillations in the TFs are evident; activation of *O* is followed by activation of *N*, which is followed by activation of *P*, then *O* and so on.

**Figure 3.**
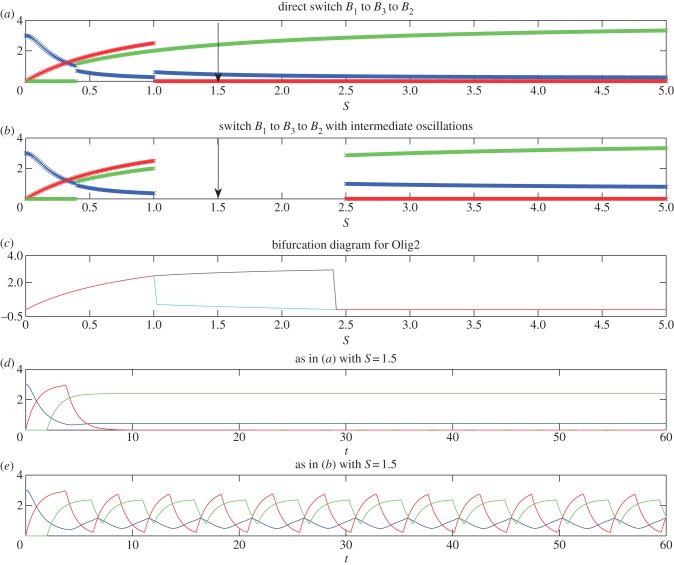
Numerical profiles of the simplified model, illustrating the existence of a sharp switch (*a*,*d*) or oscillations at intermediate S levels (*b*,*c*,*e*) in the expression profiles of Pax6 (blue), Olig2 (red) and Nkx2.2 (green) in the transition *B*_1_ → *B*_3_ → *B*_2_. Model parameters are: *α* = 3, *β* = 5, *γ* = 4, *h*_1_ = 2, *h*_2_ = 2, *O*_crit_ = 1, *P*_crit1_ = 1, *O*_crit1_ = 5, *N*_crit1_ = 2, *k*_1_ = 1, *k*_2_ = 1, *k*_3_ = 1 and in (*a*) and (*d*) *N*_crit_ = 1 and in (*b*), (*c*) and (*e*) *N*_crit_ = 2. In (*a*) and (*b*), the profiles are generated at *t* = 60, at which time the profiles have converged to steady state (to within a tiny tolerance), if they ever will do so. If they do not, we simply leave a gap in the plot. In (*c*), we show the steady state value (red line) or the maximum (black line) and minimum (cyan line) values of *O* for parameters as in (*b*), once the system has converged either to steady state or to a limit cycle (we use *t* = 500), plotted against *S*. In (*d*) and (*e*), we plot time courses of *P*, *O* and *N* for parameters as in (*a*) and (*b*), respectively, and for fixed *S*. The values of *S* used in (*d*) and (*e*) are marked with arrows in (*a*) and (*b*), respectively.

Figure 3 shows numerical simulations for parameter values corresponding to possibilities (2) and (4) from §3, indicating how the levels of the three TFs vary in response to different levels of *S* for two different parameter sets. In both cases, there is first a transition from *O* on and *N* off to co-expression of *O* and *N* as *S* increases. In figure 3*a*, the parameters satisfy *P*_max_/*P*_crit1_ < 1 + (*N*_crit1_/*N*_crit_)^*h*_1_^, so that the transition to *B*_2_ (i.e. the subsequent switching off of *O*) is direct. By contrast, in figure 3*b* the parameters satisfy *P*_max_/*P*_crit1_ > 1 + (*N*_crit1_/*N*_crit_)^*h*_1_^, so that there is an intermediate range of values of *S* for which there are temporal oscillations. In figure 3*c*, we show for the system as in figure 3*b*, either the stable steady-state values of *O* (in red), or, once the system has converged to a limit cycle, the maximum (in black) and minimum (in cyan) values of *O*. In figure 3*d*, we illustrate the temporal behaviour of the model with parameter values as in figure 3*a*, for a value of *S* above the transition from *B*_3_. This reveals the sequential activation of *P*, then *O* and finally *N*. In figure 3*e*, we illustrate the temporal behaviour of the model with parameter values as in figure 3*b*, for a level of *S* above the transition from *B*_3_ but below the transition to *B*_2_. In this case, temporal oscillations in the TFs are evident, activation of *O* is followed by *N*, which is followed by *P*, then *O*, etc.

**Figure 4.**
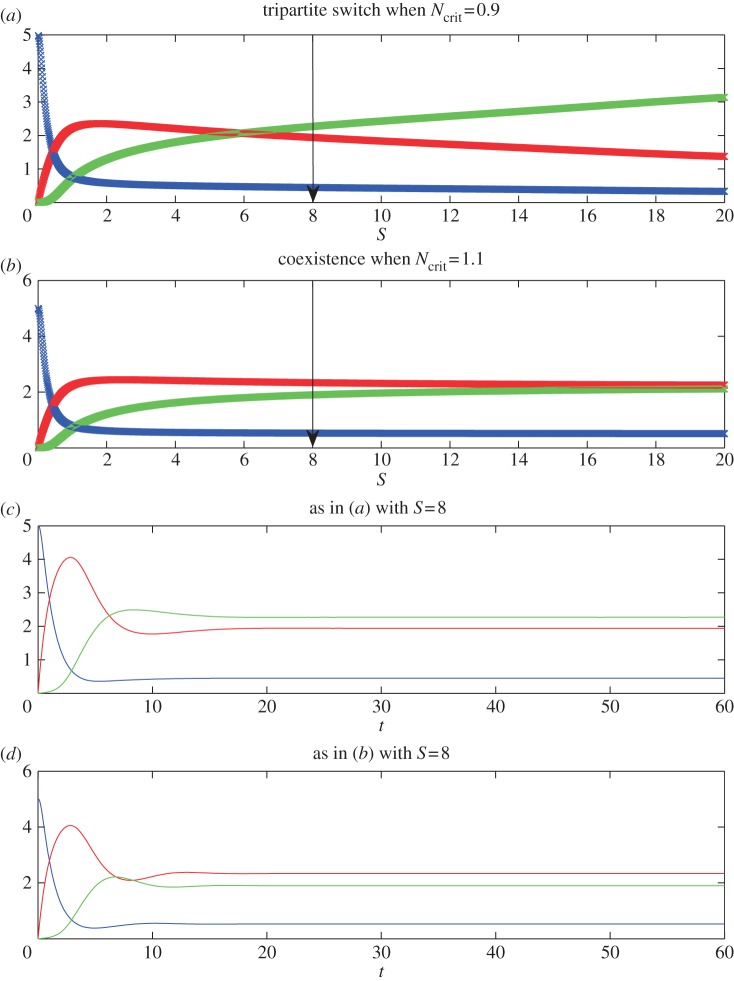
Numerical profiles of the full model, with parameters as in figure 2, but with Hill coefficients *h*_3_ = *h*_4_ = *h*_5_ = 2. In this case, as in figure 2, when *N*_crit_ = 0.9 there is a straight switch from Pax6 dominance at low Shh, to Olig2 dominance at intermediate Shh, to Nkx2.2 dominance at high Shh, although Nkx2.2 and Olig2 are coexpressed for a large range of Shh concentration (*a*,*c*). For *N*_crit_ = 1.1 (*b*,*d*), unlike the simplified model, there is no intermediate regime of oscillations, and Nkx2.2 never dominates Olig2. In (*a*) and (*b*), the profiles are generated at *t* = 60, at which time the profiles have converged to steady state (to within a tiny tolerance), if they ever will do so. In (*c*) and (*d*), we plot time courses of *P*, *O* and *N* for parameters as in (*a*) and (*b*), respectively, and for fixed *S*. The values of *S* used in (*c*) and (*d*) are marked with arrows in (*a*) and (*b*), respectively.

**Figure 5.**
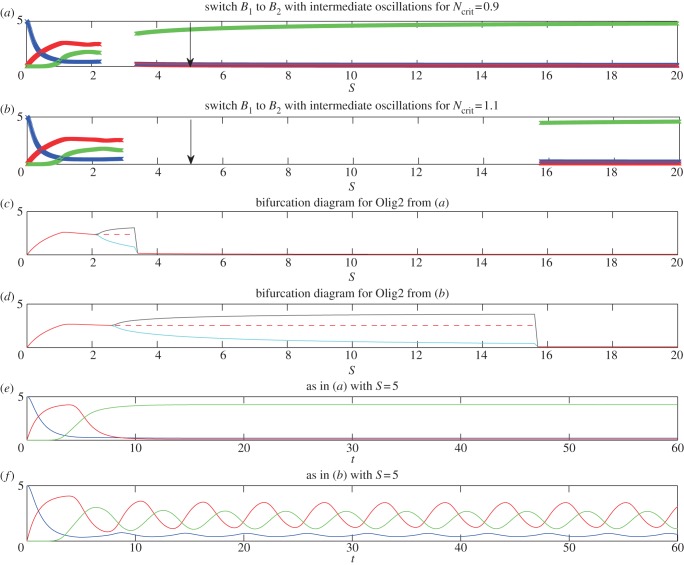
Numerical profiles of the full model, with parameters as in figure 2, but with Hill coefficients *h*_3_ = *h*_4_ = *h*_5_ = 5. In this case, as in figure 2, when *N*_crit_ = 1.1 there is an intermediate range of Shh concentrations for which the system exhibits oscillations (*c*,*d*,*f*). This range is extended in size relative to the simplified model. In addition, there is also an intermediate range of Shh concentration for which the system exhibits oscillations, when *N*_crit_ = 0.9 (see (*a*) and (*c*)). The diagrams of the steady state values (red line, solid when the state is stable and dashed when it is unstable), maximum (black line) and minimum (cyan line) values of *O*, once the system has converged to either a steady state or a limit cycle (we use *t* = 500), are shown in (*c*) for parameters as in (*a*), and in (*d*) for parameters as in (*b*). We note that there can be more than one unstable steady state, but, for clarity, we only show the continuation of the state which was stable before the bifurcation to the limit cycle. We show for comparison the temporal profiles of the TFs for *S* = 5 for *N*_crit_ = 0.9 (*e*) and *N*_crit_ = 1.1 (*f*). Only in the latter case is *S* = 5 within the range of *S* leading to oscillations. In (*a*) and (*b*), the profiles are generated at *t* = 60, at which time the profiles have converged to steady state (to within a tiny tolerance), if they ever will do so. The values of *S* used in (*e*) and (*f*) are marked with arrows in (*a*) and (*b*), respectively.

Figures 4 and 5 show simulations of the full model with Hill functions, instead of Heaviside functions describing the repression of *N* and *O* (see appendix C); the other parameters are as in figure 2. For simplicity, we consider the case when *h*_3_ = *h*_4_ = *h*_5_ = *h* and figure 4 shows the case *h* = 2, whereas figure 5 shows the case *h* = 5. In these simulations, as in those above, it is possible to observe either a switch from high *P* to high *O* to high *N* or for there to be an intermediate range of *S* for which the TFs oscillate. However, the more gradual repression functions provided by the Hill functions tend to cause transitions to occur at higher values of *S*. The effect of Hill function repression on *N* and *O* on the existence of oscillations is complex. For *h* = 2, oscillations are absent (figure 4); however, for *h* = 5 there is an extended range of values of *S* for which oscillations occur (figure 5*c*,*d*) and indeed there is a range of values of *S* for which the system, with other parameters as in figure 2*a*, shows oscillations. Moreover, for certain values of the parameters, the system displays damped oscillations. This is not possible in the simplified model using Heaviside functions, since when the steady states exist, they are stable nodes (the eigenvalues of the Jacobian matrix are −*k*_1_, − *k*_2_ and −*k*_3_). We also note that oscillations, when they exist, are simple and periodic—a proof of this for the Hill function model is given in appendix D.

It is clear from these numerical simulations that the simplified Heaviside function model accurately predicts how the system will behave for very strong repressive interactions that result in sharp cross-repression functions on *N* and *O* but, as expected, it is less accurate at recapitulating the effect of weaker repressive activities. Importantly, the generic types of behaviour displayed by the simplified model—either switches between the regimes of expression of each of the transcription factors, with hysteresis, or intermediate regimes of oscillations—are features of the system whether Heaviside or Hill functions are used to describe the repression functions. The precise ranges of the parameters for which oscillatory behaviour occurs are determined by the sharpness of the repression functions. Indeed, it is surprising how differently (in quantitative terms) the system can respond in the case *h* = 5 from the limiting case with Heaviside function repressions on *N* and *O*. Thus, the simplified model helps explain the mechanisms by which switch-like or oscillatory behaviour can arise, but without more detailed measurement of the cross-repression functions, it should not be considered a fully quantitative model of neural tube patterning.

## 5. The AC–DC signalling motif

**Figure 6.**
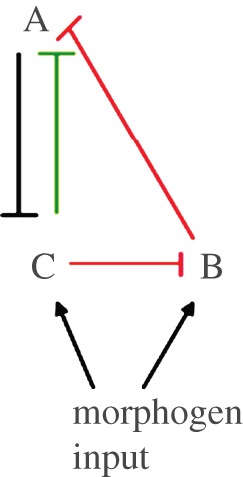
The AC–DC circuit. Depending on whether the green or the red interactions are stronger, the circuit behaves as a positive or negative feedback loop. It can either display bistability and hysteresis or oscillations.

Our analysis indicates that the gene circuit described in figure 1*a* can either behave as a three-way multistate switch—expressing *P* at low levels of *S*, *O* at intermediate levels and *N* at high levels—or it can display oscillations in the TF levels for intermediate values of *S*. It is clear from the inequalities (3.4)–(3.8) that we can let *O*_crit1_ → ∞ and not qualitatively affect the results. Thus, the repression of *N* by *O* is not necessary to the tripartite patterning or the oscillatory behaviour. It may of course have another role, for example, in attenuating noise in the system. However, for the purposes of this study, we may neglect the repression of *N* by *O*. We term the remaining gene circuit the AC–DC signalling motif (figure 6), since it is capable either of switch-like or oscillatory behaviour, depending on the parameter values.

Considering the simplified case in which the system takes route 1 and *h*_1_ = *h*_2_, oscillatory behaviour is seen if *N*_max_/*N*_crit_ < *O*_max_/*O*_crit_; otherwise, the behaviour is switch-like. The condition for oscillatory behaviour is therefore equivalent to the requirement that the repression of *P* by *O* is stronger than that of *P* by *N*. With this in mind, if we consider the motif in figure 6, the oscillatory behaviour is achieved if the red connections dominate the green one. In these cases, the circuit approximates a repressilator [28] and consists of a three-component negative feedback loop, which generically exhibits oscillations. Conversely, if the green connection is stronger than the red connections, then the circuit generates positive feedback between A and C. Positive feedback loops show bistability, switch-like behaviour and hysteresis [29]. Thus, changes in the strength of repression between the TFs would be sufficient to change the behaviour of this circuit. In this view, the AC–DC motif is a tunable positive/negative feedback loop, displaying switch-like or oscillatory behaviour.

## 6. The logic of multipartite expression

**Figure 7.**
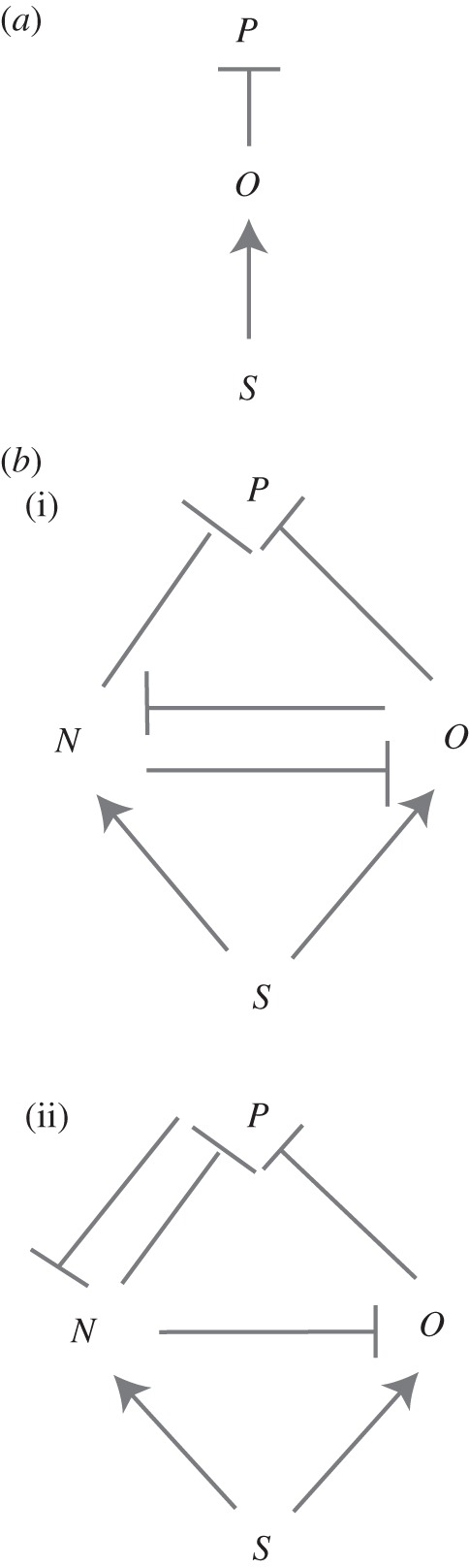
The logic of multipartite expression. A natural circuit to give rise to two domains of gene expression in response to morphogen is given in (*a*). *S* denotes the morphogen, *O* the TF expressed at high morphogen concentration and *P* the TF expressed at low morphogen concentration. (*b*) Alternative circuits that may be able to give rise to three domains of gene expression. Once again *S* denotes the morphogen, *N* is the TF expressed at high, *O* at intermediate and *P* at low morphogen concentration. As detailed in the text, (i) requires differential sensitivity of *O* and *N* to the morphogen to give three domains of TF expression. (ii) The AC–DC circuit, which can give rise to three domains of gene expression for a wide range of parameters.

The AC–DC signalling motif may be the natural design for a transcriptional network involved in tissue patterning. The regulatory logic of such a network should encode spatial domains of gene expression in response to appropriate cues. In the simplest case of a morphogen that specifies just two domains of gene expression (the precursors to two types of differentiated cells), then an obvious network design that is sufficient to transform a graded signal into two distinct regions of gene expression is given in figure 7*a*. Explicitly, once the level of signal produced by the morphogen passes a threshold it induces one of the TFs (which we label *O*). This TF then represses a second (which we label *P*) that is expressed in the field of cells independent of the morphogen. Hence, at high concentrations of the morphogen, *O* alone is expressed whereas at low concentrations, *P* alone is expressed.

An intuitive logic then allows the extension of the simple case to specify three gene expression domains. Label the TFs *N* (expressed at high morphogen concentration), *O* (expressed at intermediate morphogen concentration) and *P* (expressed at low morphogen concentration). First, *N* needs to be induced by the morphogen, necessitating an activating link from the morphogen (*S*) to *N*. Second, since *O* is no longer expressed at high morphogen concentration, it should be repressed by *N*. Third, in order that *P* is not switched back on at high concentrations when *O* is repressed, *N* should repress *P*. Finally, in order that *N* is not switched on at intermediate values of the morphogen, it should be repressed by either *P* or *O*. Including these interactions gives us two possible networks which may be capable of specifying three domains of gene expression. They are displayed in figure 7*b*(i) and (ii). The topology shown in figure 7*b*(i) is symmetric with respect to *O* and *N*. This means that in order for the symmetry to be broken and *O* to be expressed at intermediate morphogen concentrations while *N* is expressed at high concentrations, there must be quantitative differences in the sensitivities of *N* and *O* to *S* and in the cross-repression parameters. Thus, we would expect this to work only if *O* is more sensitive to the morphogen, but *N*, once expressed, represses *O* more strongly than *O* represses *N*. In this case, it is relatively simple to analyse the network, since *P* has no influence on *N* and *O*. The system of ODEs therefore decouples and we can first solve for *N* and *O* in terms of *S*, as is done in [17] (see also [18]). In the simplified model of §2, it is straightforward to see that the network in figure 7*b*(i) cannot give rise to three domains of gene expression, since, if *O* represses *N* at intermediate concentrations, it will continue to do so at high concentrations. In the full model, it is possible for three domains of gene expression to arise with the network in figure 7*b*(i), but the parameters have to be carefully chosen, such that *O* is very slightly more sensitive to the morphogen, while *N* represses *O* more strongly than *O* does *N*. This sensitivity to parameters suggests that it might be biologically less plausible. For instance, it might not be biologically feasible to tune the parameters, perhaps because the TFs are used in other processes that constrain their flexibility. This would make it unlikely for the motif to be discovered by evolution or maintained if it were adopted. In addition, the parameter sensitivity might result in a system that lacks robustness and is prone to degradation by the noise inherent in biological processes.

By contrast, the second circuit (figure 7*b*(ii)) does not suffer from the same disadvantages. This topology corresponds to that of the AC–DC network, which we have shown above can give rise either to three domains of gene expression (with hysteresis near the boundaries) or oscillations for intermediate ranges of the morphogen concentration. Since the three-domain multistate switch behaviour of this circuit is the outcome for a wide range of parameter values, we conclude that the AC–DC network has a very natural topology to give rise to tripartite gene expression. It appears to be a more plausible mechanism that could be produced by evolution and once adopted it is sufficiently flexible to be retained during subsequent natural selection. We note that the actual network of morphogen and transcription factors involved in ventral neural tube patterning is a superposition of the networks in figure 7*b*(i) and (ii). However, as we noted in §3, the repression of Nkx2.2 by Olig2 seems to be unimportant in the patterning process, at least at this simple level of analysis, which neglects noise and time delays. Nevertheless, we expect that there will exist alternative network designs which will be capable of interpreting the morphogen.

**Figure 8.**
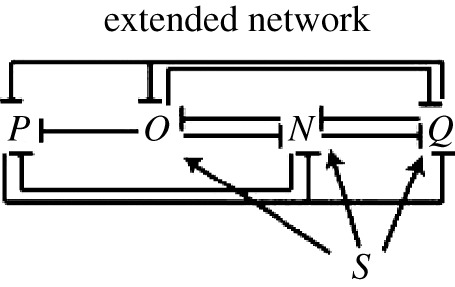
A diagram of a network capable of generating four stripes of gene expression. *Q* is expressed at the highest levels of the morphogen *S*, followed by *N*, followed by *O*, while *P* is expressed at the lowest levels.

The AC–DC network topology can be further extended, using similar logic, to produce four or more domains of gene expression. To extend a three domain network to four domains by adding a TF, we term *Q*, to be expressed at a higher morphogen concentration than *N*: *Q* should be induced by the morphogen and should repress the other three TFs, ensuring none of them is expressed at the highest concentrations of morphogen. In order to make the network robust, *P* should repress *Q*. In addition, to prevent *Q* from being expressed at concentrations of the morphogen below its threshold, *Q* should be repressed by *N* and/or *O*. Similar to the case in figure 7*b*(i), if *N* represses *Q* but *O* does not, then the network topology is symmetric with respect to *N* and *Q*, so that only quantitative differences can cause the asymmetry in expression. However, if *Q* is repressed by *N*, *O* and *P*, this produces a circuit (figure 8) capable of robustly producing four domains of gene expression. Thus, although the reasoning becomes increasingly complicated, the same mechanism can be used to generate multiple switches in gene expression.

## 7. Discussion

Understanding the mechanisms that pattern embryonic tissues is a central question of developmental biology. In most embryonic tissues, initially homogeneous fields of cells become subdivided into distinct regions each of which expresses a different set of genes. One way in which this patterning process is controlled relies on morphogens—graded signals—that specify different gene expression domains as a function of the level of signalling. Thus, morphogens must effect a multistate switch in the developing tissue. While much attention has focused on the mechanism of graded signalling and the consequences of this for the precision and robustness of tissue patterning, less consideration has been given to how the multistate switch is implemented. In this study, motivated by empirical data from the vertebrate neural tube, we provide evidence that the activity of a gene regulatory circuit, which connects three TFs through a set of repressive interactions, provides a reliable and adaptable multiway switch. We term this circuit the AC–DC motif. Our analysis suggests that the mechanism that underpins this motif offers a natural way to achieve a multistate switch as it provides the robustness and flexibility demanded by an evolving biological system. We further show that the regulatory logic of the AC–DC motif can be used to extend the switch to produce additional states each of which depends on different levels of signalling. Together these features suggest that this mechanistic strategy might be employed in other developing tissues patterned by morphogens.

In addition to morphogen gradients, temporal oscillations are deployed in developing tissues to produce pattern. In particular, the presence of oscillations in a growing field of cells can be used to generate recurrent gene expression patterns and structures [30,31]. Moreover, oscillations in TFs are central to various basic cellular functions [32–35]. Strikingly, our analysis indicates that, in addition to a multiway switch, the AC–DC circuit is capable of producing oscillations in TF expression in response to a defined range in the level of the signal, and we define the kinetic parameters responsible for selecting between oscillations and a multistate switch. The ability of the AC–DC motif to generate oscillations as well as a multistate switch raises the possibility that this circuit, or closely related versions, are employed to generate oscillations in developing tissues. For instance, it is notable that gradients of Wnt and FGF signalling are present during somitogenesis and the oscillations that are responsible for somite formation are produced in regions of tissue exposed to specific ranges of concentrations [1]. Thus, it is conceivable that an AC–DC motif is deployed downstream of graded signalling to generate the oscillations necessary for somitogenesis.

Whether the AC–DC motif produces oscillations or a multistate switch is determined by the strength of specific repressive interactions within the circuit and there is a continuum of parameter values that link these two types of behaviour. Thus, it is possible to transition between a multistate switch and oscillations by gradually adjusting the relevant parameters. This raises the intriguing possibility that the AC–DC motif might have first arisen during the course of evolution as either an oscillator or a multiway switch. Then during subsequent natural selection the circuit might have been co-opted to generate the alternative behaviour. Among the parameters that distinguish oscillatory and multistate switch behaviour are *O*_crit_ and *N*_crit_ (compare for example figure 5*a*,*c* and 5*b*, *d*). An increase in these parameters results in a requirement for increased concentrations of *O* and *N* to repress their target gene. An obvious biological correlate to this change is an alteration in the binding affinity of the TFs for their target sites. *In vivo*, this is determined by a combination of factors, including the sequence of DNA bound by the TF and the presence of protein co-factors that interact with the TF. Hence, changes in the DNA sequence of the relevant binding sites in the enhancer of the target genes that alter binding affinity provide one plausible mechanism by which a change in behaviour of an AC–DC circuit could be achieved. Alternatively, modification in the expression or function of co-factors during evolution could also produce a change from oscillation to a multiway switch. In this case, the differential expression of co-factors in different tissues, or at different developmental stages, would allow the AC–DC motif to be deployed within the same species in both oscillatory and multiway switch form. This versatility suggests that the AC–DC motif is an attractive circuit that once discovered would provide a substrate that could be modified and redeployed repeatedly to perform different tasks during subsequent evolution.

Models which can display oscillations or switch-like behaviour have been presented before for chemical systems. These include the Belousov–Zhabotinsky chemical reactions [36] and the hypothetical system of reactions named the Brusselator [37] (see [38] for a review). In addition, hypothetical gene regulatory circuits with structures resembling the AC–DC motif, albeit lacking the input signal, have been considered [20,39].^2^ Yang *et al.* focused on the oscillatory behaviour of the network within single cells (it is capable of two types of oscillation: relaxation oscillations and repressilator-style oscillations). The analysis led to the conclusion that oscillations are feasible even when positive feedback dominates in the network (where we predict bistability), if there is a separation of time scales. The relaxation oscillations produced in this way are possible in the specific example that we analyse, if the dynamics of *O* are much slower than *N* and *P*. Although this is conceivable, there is no experimental evidence to support the idea that the dynamics of the different TFs differ markedly and it would require, for example, that the half-life of *O* differs considerably from *N* or *P*. Yang *et al.* also show that the motif is capable of producing two stable steady states if cells are coupled together through one of the factors. This is interpreted as a mechanism leading to stable ‘differentiation’, but it would require the movement of one of the TFs between cells. An alternative, perhaps more biologically plausible, possibility demonstrated by our analysis is that the topology of the network allows either oscillations or a multiway switch in individual cells as a function of the strength of repressive interaction within the circuit. Importantly, we show how the level of an input signal can provide a spatially varying parameter in the dynamical system that allows a three-way switch or spatially localized oscillations in a tissue. In general, motifs like the AC–DC motif illustrate that knowledge of the topology of a circuit is not sufficient to understand its qualitative dynamic behaviour and that detailed quantitative analysis coupled with experiments are required to fully explore its potential (see [38,40] for further examples).

In summary, we have presented a model of gene regulation derived from empirical observations of the patterning of progenitor cells in the neural tube. The model is based on the morphogen control of a network of TFs. A simplification involving the assumption that some of the regulatory interactions are threshold-like allowed the analysis of the model dynamics. This revealed that, depending on the parameter values, the network produces sharp switches from one gene expression domain to another (with hysteresis) as the morphogen concentration changes or the network generates oscillations for specific values of the morphogen. We show these alternative behaviours result from a tunable dominance of either positive or negative feedback between the TFs. We argue that the logic of the circuit makes it a natural motif to use to produce stripes of gene expression and that this strategy could be re-used during the course of evolution to effect either differential spatial patterns or oscillations in gene expression.

In future, we intend to investigate the noise transmission properties of the AC–DC motif and the Shh system. The morphogen concentration seen by each cell contains temporal fluctuations and transcription, translation and molecular degradation are noisy processes and yet the boundaries between gene expression domains are sharp. In addition, the repression of Nkx2.2 by Olig2 does not seem necessary for the deterministic dynamical properties of the system. We would like to investigate whether it has an effect on the noise transmission. In addition, we are interested in the robustness of the formation of the Shh gradient [41] and in the interaction between gradient formation and interpretation [42].

## Endnotes

1 Since we assume that *N* and *O* expressions are dependent on *S*, they must be low at low *S*. However, to be consistent with the biological data, we demand that *N* does not increase immediately as *S* increases. Thus, low but non-zero *S* will give rise to high *P*, low *O* and no *N*. It is also inevitable, from the continuous repression of *P* by *N* and *O*, that if either *N* or *O* is very large, *P* will be low.

2 We note that Yang *et al.* use a different form of repression function from that in our model. In the second equation of (3) in [20], the production rate of ‘*v*’ consists of a sum of two decreasing Hill functions. This means that both repressors must have a high concentration to repress the controlled gene ‘*v*’ to low levels. In the neural tube, it appears that a high concentration of either Nkx2.2 or Olig2 is sufficient to repress Pax6 and similarly a high concentration of either Pax6 or Olig2 is sufficient to repress Nkx2.2 to low levels. Thus, we use the repression functions in the forms indicated in equations (C 1)–(C 3).

## Appendix A. Derivation of conditions (3.4)–(3.6) for biologically realistic routes through the steady states to be taken

The condition for *B*_1_ to be stable at low signal is: as *S* → 0, condition (a) in §3 becomes

so

A1

The condition for *B*_2_ to be stable at high signal is: as *S* → ∞, condition (b) in §3 becomes

so

A2

Furthermore, in order to make the transition out of *B*_1_, *B*_1_ should become unstable as *S* is increased. This means that there exists *S* such that

The former condition gets harder to satisfy as *S* increases, whereas the latter condition gets easier to satisfy. Therefore, increasing *S* can only cause a transition out of *B*_1_ by starting to satisfy the latter condition, while the former condition still holds. Therefore, such a transition will occur when

provided this can occur, i.e. *α* < *k*_1_*P*_crit1_[1 + (*β*/*O*_crit_*k*_2_)^*h*_2_^], and provided *β*(*S*) < *k*_2_*O*_crit1_ when it does, i.e. provided (*α*/*k*_1_*P*_crit1_ − 1)^1/*h*_2_^ *O*_crit_ < *O*_crit1_.

Thus, the transition out of *B*_1_ occurs if and only if

A3

The conditions for *B*_2_ to exist get easier to satisfy as *S* increases, so once the transition to *B*_2_ is made, the system should stay in *B*_2_. The transition out of *B*_1_ however can be to *B*_3_ or to a state in which no steady state is stable. In this case, we need to exclude the possibility that the system transitions back to *B*_1_, by satisfying the first condition of criterion (a) in §3, before it can transition to *B*_2_. Such a transition should again be permanent, since the first condition of criterion (a) in §3 becomes easier to satisfy as *S* increases. We note that states *B*_2_ and *B*_3_ are mutually exclusive (), so our condition in equation (A 2) ensures that the system cannot stay in *B*_3_ as *S* → ∞.

Given the conditions established so far, a transition back to *B*_1_ (by satisfying the first condition of criterion (a)) in §3 occurs before a transition to *B*_2_ if and only if  is satisfied before (i.e. for a lower value of *S*) both  and  are satisfied. That is, if either

or

i.e. either

or

Thus, the final conditions for one of the biologically relevant routes through states to occur are

A4

The first and third conditions of criterion (c) in §3 are automatically satisfied at the transition out of *B*_1_. So (given the previous conditions) transition to *B*_3_ occurs if and only if

if and only if

if and only if

if and only if

A5

If this condition is not satisfied, then the system can never transition to *B*_3_, since  gets harder to satisfy as *S* increases.

(We note that if we want high *S* to correspond to *S* taking a finite maximal value, rather than ∞, we simply re-scale *β* and *γ*.)

## Appendix B. Threshold values of Shh concentration at transitions

Let *u* = *S*/(1 + *S*), so that *S* = *u*/(1 − *u*).

The transition out of the state *B*_1_ occurs when

B1

In the case of a straight transition into *B*_2_ this is clearly also the value of *u* for transition into *B*_2_. If instead there are oscillations followed by a transition into *B*_2_, this later transition occurs when

B2

In the case where there is a transition from *B*_1_ to *B*_3_, this always occurs for *u* as in equation (B 1). The subsequent transition out of *B*_3_ occurs for

B3

This can either lead directly to *B*_2_ or to oscillations. In the latter case, the final transition from oscillations to *B*_2_ occurs for *u* as in equation (B 2).

## Appendix C. Full model equations

The full model equations, including Hill function repression on *N* and *O*, are given by

C1

C2

and

C3

where *h*_3_ is the Hill coefficient determining how sharp the repression of *O* by *N* is, *h*_4_ is the Hill coefficient determining how sharp the repression of *N* by *O* is and *h*_5_ is the Hill coefficient determining how sharp the repression of *N* by *P* is. The other parameters are as in §2.

## Appendix D. Demonstration that a Poincaré–Bendixson-type result holds for the system with Hill function repressions

We consider the full model equations (C1)–(C3), with *S* fixed for . We write *x* = (*P, O, N*) and

We note that the region (0,*P*_max_) × (0,*O*_max_) × (0,*N*_max_) is forward invariant. It is also convex and hence *p*-convex (where , *x* ≤ *y* if and only if *x*_*i*_ ≤ *y*_*i*_, *i* = 1,2,3 and a set *U* is *p*-convex whenever  and *x* ≤ *y* implies that the straight line between *x* and *y* belongs to *U*).

We also note that ((0,*P*_max_) × (0,*O*_max_) × (0,*N*_max_)) and the system is competitive in (0,*P*_max_) × (0,*O*_max_) × (0,*N*_max_), since

, since *P* does not influence the level of *O*. The other entries of *D*_*x*_*f* are strictly negative on (0,*P*_max_) × (0,*O*_max_) × (0,*N*_max_). This means that *D*_*x*_*f* is irreducible on (0,*P*_max_) × (0,*O*_max_) × (0,*N*_max_).

The system thus satisfies the conditions for theorem 2.2 in [43], which says ‘Let *L* be a compact *α* or *ω* limit set of an irreducible cooperative or competitive system in ℝ^3^. If *L* contains no equilibria then *L* is a closed orbit.’

Thus, there is a Poincaré–Bendixson-type result for this system; therefore, if the system converges towards oscillations, these must be periodic and non-chaotic.

## References

[1] Pourquie O 2001 Vertebrate somitogenesis. Annu. Rev. Cell Dev. Biol. 17, 311–350 10.1146/annurev.cellbio.17.1.311 (doi:10.1146/annurev.cellbio.17.1.311) 11687492

[2] Pages F Kerridge S 2000 Morphogen gradients: a question of time or concentration? Trends Genet. 16, 40–44 10.1016/S0168-9525(99)01880-6 (doi:10.1016/S0168-9525(99)01880-6) 10637630

[3] Dessaud E Yang LL Hill K Cox B Ulloa F Ribeiro A Mynett A Novitch BG Briscoe J 2007 Interpretation of the Sonic Hedgehog morphogen gradient by a temporal adaptation mechanism. Nature 450, 717–721 10.1038/nature06347 (doi:10.1038/nature06347) 18046410

[4] Briscoe J Ericson J 2001 Specification of neuronal fates in the vertebrate neural tube. Curr. Opin. Neurobiol. 11, 43–49 10.1016/S0959-4388(00)00172-0 (doi:10.1016/S0959-4388(00)00172-0) 11179871

[5] Briscoe J Novitch BG 2008 Regulatory pathways linking progenitor patterning, cell fates and neurogenesis in the ventral neural tube. Proc. Trans. R. Soc. B 363, 57–70 10.1098/rstb.2006.2012 (doi:10.1098/rstb.2006.2012) PMC2605486 17282991

[6] Casali A Struhl G 2004 Reading the Hedgehog morphogen gradient by measuring the ratio of bound to unbound Patched protein. Nature 431, 76–80 10.1038/nature02835 (doi:10.1038/nature02835) 15300262

[7] Jacob J Briscoe J 2003 Gli proteins and the control of spinal-cord patterning. EMBO Rep. 4, 761–765 10.1038/sj.embor.embor896 (doi:10.1038/sj.embor.embor896) 12897799 PMC1326336

[8] Bai CB Stephen D Joyner AL 2004 All mouse ventral spinal cord patterning by Hedgehog is Gli dependent and involves activator function of Gli3. Dev. Cell 6, 103–115 10.1016/S1534-5807(03)00394-0 (doi:10.1016/S1534-5807(03)00394-0) 14723851

[9] Balaskas N Ribes V Panovska J Sasai N Ribeiro A Page KM Briscoe J Dessaud E 2012 Morphogen gradient interpretation as a systems level property of a gene regulatory network. Cell 148, 273–284 10.1016/j.cell.2011.10.047 (doi:10.1016/j.cell.2011.10.047) 22265416 PMC3267043

[10] Thomas R d'Ari R 1990 Biological feedback. Boca Raton, FL: CRC Press

[11] Thomas R Thieffry D Kauffman M 1995 Dynamical behavior of biological regulatory networks. I. Biological role of feedback loops and practical use of the concept of the loop-characteristic state. Bull. Math. Biol. 57, 247–276 10.1007/BF02460618 (doi:10.1007/BF02460618) 7703920

[12] Smolen P Baxter DA Byrne JH 2000 Modeling transcriptional control in gene networks—methods, recent results and future directions. Bull. Math. Biol. 62, 247–292 10.1006/bulm.1999.0155 (doi:10.1006/bulm.1999.0155) 10824430

[13] Smolen P Baxter DA Byrne JH 2000 Mathematical modeling of gene networks. Neuron 26, 567–580 10.1016/S0896-6273(00)81194-0 (doi:10.1016/S0896-6273(00)81194-0) 10896154

[14] Hasty J McMillen D Collins JJ 2002 Engineered gene circuits. Nature 420, 224–230 10.1038/nature01257 (doi:10.1038/nature01257) 12432407

[15] Tyson J Othmer HG 1978 The dynamics of feedback control circuits in biochemical pathways. Prog. Theor. Biol. 5, 2–62

[16] Goodwin BC 1965 Oscillatory behavior of enzymatic control processes. Adv. Enzyme Reg. 3, 425–439 10.1016/0065-2571(65)90067-1 (doi:10.1016/0065-2571(65)90067-1) 5861813

[17] Cherry JL Adler FR 2000 How to make a biological switch. J. Theoret. Biol. 203, 117–133 10.1006/jtbi.2000.1068 (doi:10.1006/jtbi.2000.1068) 10704297

[18] Saka Y Smith JC 2007 A mechanism for the sharp transition of morphogen gradient interpretation of *Xenopus*. BMC Dev. Biol. 7, 47–56 10.1186/1471-213X-7-47 (doi:10.1186/1471-213X-7-47) 17506890 PMC1885807

[19] Smith H 1987 Oscillations and multiple steady states in a cyclic gene model with repression. J. Math. Biol. 25, 169–190 10.1007/BF00276388 (doi:10.1007/BF00276388) 3611980

[20] Yang D Li Y Kuznetsov A 2009 Characterization and merger of oscillatory mechanisms in an artificial gene regulatory network. Chaos 19, 033115 10.1063/1.3176943 (doi:10.1063/1.3176943) 19791995

[21] McAdams H Shapiro L 1995 Circuit simulation of genetic networks. Science 269, 650–656 10.1126/science.7624793 (doi:10.1126/science.7624793) 7624793

[22] Glass L Kauffman SA 1973 The logical analysis of continuous, non-linear biochemical control networks. J. Theoret. Biol. 39, 103–129 10.1016/0022-5193(73)90208-7 (doi:10.1016/0022-5193(73)90208-7) 4741704

[23] de Jong H Gouze J-L Hernandez C Page M Sari T Geiselmann J 2004 Qualitative simulation of genetic regulatory networks using piecewise-linear models. Bull. Math. Biol. 66, 301–340 10.1016/j.bulm.2003.08.010 (doi:10.1016/j.bulm.2003.08.010) 14871568

[24] Casey R de Jong H Gouze JL 2006 Piecewise-linear models of genetic regulatory networks: equilibria and their stability. J. Math. Biol. 52, 27–56 10.1007/s00285-005-0338-2 (doi:10.1007/s00285-005-0338-2) 16195929

[25] Briscoe J Pierani A Jessell TM Ericson J 2000 A homeodomain protein code specifies progenitor cell identity and neuronal fate in the ventral neural tube. Cell 101, 435–445 10.1016/S0092-8674(00)80853-3 (doi:10.1016/S0092-8674(00)80853-3) 10830170

[26] Novitch BG Chen AI Jessell TM 2001 Coordinate regulation of motor neuron subtype identity and pan-neuronal properties by the bHLH repressor Olig2. Neuron 31, 773–789 10.1016/S0896-6273(01)00407-X (doi:10.1016/S0896-6273(01)00407-X) 11567616

[27] Ericson J Rashbass P Schedl A Brenner-Morton S Kawakami A van Heyningen V Jessell TM Briscoe J 1997 Pax6 controls progenitor cell identity and neuronal fate in response to graded Shh signaling. Cell 90, 169–180 10.1016/S0092-8674(00)80323-2 (doi:10.1016/S0092-8674(00)80323-2) 9230312

[28] Elowitz MB Leibler S 2000 A synthetic oscillatory network of transcriptional regulators. Nature 403, 335–338 10.1038/35002125 (doi:10.1038/35002125) 10659856

[29] Gardner TS Cantor CR Collins JJ 2000 Construction of a genetic toggle switch in Escherichia coli. Nature 403, 339–342 10.1038/35002131 (doi:10.1038/35002131) 10659857

[30] Palmeirim I Henrique D Ish-Horowicz D Pourquie O 1997 Avian hairy gene expression identifies a molecular clock linked to vertebrate segmentation and somitogenesis. Cell 91, 639–648 10.1016/S0092-8674(00)80451-1 (doi:10.1016/S0092-8674(00)80451-1) 9393857

[31] Sarrazin AF Peel AD Averof M 2012 A segmentation clock with two-segment periodicity in insects. Science 336, 338–341 10.1126/science.1218256 (doi:10.1126/science.1218256) 22403177

[32] Novak B Tyson JJ 1993 Numerical analysis of a comprehensive model of M-phase control in Xenopus oocyte extracts and intact embryos. J. Cell Sci. 106, 1153–1168 8126097 10.1242/jcs.106.4.1153

[33] Goldbeter A 1995 A model for circadian oscillations in the *Drosophila* period protein (PER). Proc. R. Soc. Lond. B 261, 319–324 10.1098/rspb.1995.0153 (doi:10.1098/rspb.1995.0153) 8587874

[34] Ruoff P Rensing L 1996 The temperature-compensated Goodwin model simulates many circaidian clock properties. J. Theoret. Biol. 179, 275–285 10.1006/jtbi.1996.0067 (doi:10.1006/jtbi.1996.0067)

[35] Goldbeter A 2002 Computational approaches to cellular rhythms. Nature 420, 238–245 10.1038/nature01259 (doi:10.1038/nature01259) 12432409

[36] Field RJ Koros E Noyes RM 1972 Oscillations in chemical systems: II. Thorough analysis of temporal oscillations in the bromate–cerium–malonic acid system. J. Am. Chem. Soc. 94, 8649–8664 10.1021/ja00780a001 (doi:10.1021/ja00780a001)

[37] Prigogine I Lefever R 1968 Symmetry breaking instabilities in dissipative systems. II. J. Chem. Phys. 48, 1695–1700 10.1063/1.1668896 (doi:10.1063/1.1668896)

[38] Tyson JJ Albert R Golbeter A Ruoff P Sible J 2008 Biological switches and clocks. J. R. Soc. Interface 5(Suppl. 1), S1–S8 10.1098/rsif.2008.0179.focus (doi:10.1098/rsif.2008.0179.focus) PMC2706456 18522926

[39] Li W Krishna S Piglotti S Mitarai N Jensen MH 2012 Switching between oscillations and homeostasis in competing negative and positive feedback motifs. J. Theoret. Biol. 307, 205–210 10.1016/j.jtbi.2012.04.011 (doi:10.1016/j.jtbi.2012.04.011) 22762992

[40] Conrad E Mayo AE Ninfa AJ Forger DB 2008 Rate constants rather than biochemical mechanism determine behavior of genetic clocks. J. R. Soc. Interface 5(Suppl. 1), S9–S15. 10.1098/rsif.2008.0046.focus (doi:10.1098/rsif.2008.0046.focus) PMC2706451 18426770

[41] Saha K Schaffer DV 2005 Signal dynamics in Sonic Hedgehog tissue patterning. Development 133, 889–900 10.1242/dev.02254 (doi:10.1242/dev.02254) 16452094

[42] Jaeger J Irons D Monk N 2008 Regulative feedback in pattern formation: towards a general relativistic theory of positional information. Development 135, 3175–3183 10.1242/dev.018697 (doi:10.1242/dev.018697) 18776142

[43] Smith HL 1985 Periodic orbits of competitive and cooperative systems. J. Differ. Equ. 65, 361–373 10.1016/0022-0396(86)90024-0 (doi:10.1016/0022-0396(86)90024-0)

